# Guidance for industry: patient-reported outcome measures: use in medical product development to support labeling claims: draft guidance

**DOI:** 10.1186/1477-7525-4-79

**Published:** 2006-10-11

**Authors:** U.S. Department of Health and Human Services FDA Center for Drug Evaluation and Research, U.S. Department of Health and Human Services FDA Center for Biologics Evaluation and Research, U.S. Department of Health and Human Services FDA Center for Devices and Radiological Health

**Affiliations:** 1 U.S. Department of Health and Human Services, Food and Drug Administration, Center for Drug Evaluation and Research, Food and Drug Administration, 5600 Fishers Lane, Rockville, MD 2085, USA; 2 U.S. Department of Health and Human Services, Food and Drug Administration, Center for Biologics Evaluation and Research, Food and Drug Administration, 1401 Rockville Pike, Rockville, MD 20852-1448, USA; 3 U.S. Department of Health and Human Services, Food and Drug Administration, Center for Devices and Radiological Health, Food and Drug Administration, 1350 Piccard Drive, Rockville, MD 20850-430, USA

## Abstract

This guidance describes how the FDA evaluates patient-reported outcome (PRO) instruments used as effectiveness endpoints in clinical trials. It also describes our current thinking on how sponsors can develop and use study results measured by PRO instruments to support claims in approved product labeling (see appendix point 1). It does not address the use of PRO instruments for purposes beyond evaluation of claims made about a drug or medical product in its labeling. By explicitly addressing the review issues identified in this guidance, sponsors can increase the efficiency of their endpoint discussions with the FDA during the product development process, streamline the FDA's review of PRO endpoint adequacy, and provide optimal information about the patient's perspective of treatment benefit at the time of product approval.

A PRO is a measurement of any aspect of a patient's health status that comes directly from the patient (i.e., without the interpretation of the patient's responses by a physician or anyone else). In clinical trials, a PRO instrument can be used to measure the impact of an intervention on one or more aspects of patients' health status, hereafter referred to as PRO concepts, ranging from the purely symptomatic (response of a headache) to more complex concepts (e.g., ability to carry out activities of daily living), to extremely complex concepts such as *quality of life*, which is widely understood to be a multidomain concept with physical, psychological, and social components. Data generated by a PRO instrument can provide evidence of a treatment benefit from the patient perspective. For this data to be meaningful, however, there should be evidence that the PRO instrument effectively measures the particular concept that is studied. Generally, findings measured by PRO instruments may be used to support claims in approved product labeling if the claims are derived from adequate and well-controlled investigations that use PRO instruments that reliably and validly measure the specific concepts at issue.

The glossary defines many of the terms used in this guidance. In particular, the term *instrument *refers to the actual questions or items contained in a questionnaire or interview schedule along with all the additional information and documentation that supports the use of these items in producing a PRO measure (e.g., interviewer training and instructions, scoring and interpretation manual). The term *conceptual framework *refers to how items are grouped according to subconcepts or *domains *(e.g., the item *walking without help *may be grouped with another item, *walking with difficulty*, within the domain of *ambulation*, and *ambulation *may be further grouped into the concept of *physical ability*).

FDA's guidance documents, including this guidance, do not establish legally enforceable responsibilities. Instead, guidance documents describe the Agency's current thinking on a topic and should be viewed only as recommendations, unless specific regulatory or statutory requirements are cited. The use of the word *should *in Agency guidance documents means that something is suggested or recommended but not required.

First publication of the Draft Guidance by the Food and Drug Administration- February 2006.

## 1. Background

PRO instruments provide a means for measuring treatment benefits by capturing concepts related to how a patient feels or functions with respect to his or her health or condition. The concepts, events, behaviors, or feelings measured by PRO instruments can be either readily observed or verified (e.g., walking) or can be non-observable, known only to the patient and not easily verified (e.g., feeling depressed). Although an assessment of symptom improvement or pertinent function depends on patient perception, historically these assessments were often made by physicians who observed and interacted with patients (depression scales, heart failure severity scales, activities of daily living scales). Increasingly, such assessments are based on PRO instruments. The purpose of this guidance is to explain how the FDA evaluates such instruments for their usefulness in measuring and characterizing the benefit of medical product treatment.

The amount and kind of evidence that the FDA expects to support a labeling claim measured by a PRO instrument is the same as that required for any other labeling claim (see appendix point 2). As with other labeling claims, the determination of whether the PRO instrument supports an effectiveness endpoint includes an assessment of the ability of the PRO instrument to measure the claimed treatment benefit and is specific to the intended population and to the characteristics of the condition or disease treated. Endpoints measured by PRO instruments are most often used in support of claims that refer to a patient's symptoms or ability to function.

Note, however, that PRO instruments that measure a simple concept may not be adequate to substantiate a more complex claim. For example, PRO-based evidence of improved symptoms alone generally is not sufficient to substantiate a claim related to improvement in a patient's ability to function or the patient's psychological state. Rather, to substantiate such a general claim, a sponsor should develop evidence to show not only a change in symptoms, but how that change translates into other specific endpoints such as ability to perform activities of daily living, or improved psychological state. Accordingly, many PRO instruments are specifically designed to assess both symptoms and other possible consequences of treatment.

## 2. Patient-reported outcomes – regulatory perspective

### 2.1 Why use patient-reported outcome instruments in medical product development?

PRO instruments are included in clinical trials for new medical products because (1) some treatment effects are known only to the patient; (2) there is a desire to know the patient perspective about the effectiveness of a treatment; or (3) systematic assessment of the patient's perspective may provide valuable information that can be lost when that perspective is filtered through a clinician's evaluation of the patient's response to clinical interview questions.

#### 2.1.1 Some treatment effects are known only to the patient

For some treatment effects, the patient is the only source of data. For example, pain intensity and pain relief are the fundamental measures used in the development of analgesic products. There are no observable or physical measures for these concepts.

#### 2.1.2 Patients provide a unique perspective on treatment effectiveness

PRO instruments can be developed to measure what patients want and expect from their treatment and what is most important to them. When used to measure study endpoints, PRO instruments can augment what is known about the product based on the clinician perspective or physiologic measures. This is important because improvements in clinical measures of a condition may not necessarily correspond to improvements in how the patient functions or feels. For example, clinically meaningful improvements in lung function as measured by spirometry may not correlate well with improvements in asthma-related symptoms and their impact on a patient's ability to perform daily activities.

#### 2.1.3 Formal assessment may be more reliable than informal interview

Seeking information from patients about their symptoms and the impact of those symptoms on function is not new. In clinical practice, to obtain information known only to the patients, clinicians often assess patient status by informally asking questions such as, "How many pillows do you sleep on?" or, "Do you cough at night?" In clinical trials, clinical assessments are formalized using specific questions because a structured interview technique minimizes measurement error and ensures consistency. Self-completed questionnaires that are given directly to patients without the intervention of clinicians are often preferable to the clinician-administered interview and rating. Self-completed questionnaires capture directly the patient's perceived response to treatment, without a third party's interpretation, and may be more reliable than observer-reported measures because they are not affected by interobserver variability (which usually can be reduced only by extensive training of observers). On the other hand, PRO measures may be affected by interpatient variability if the instrument is not easily understood and completed by patients. Despite these concerns, well-developed and adequately validated PRO instruments have been shown to give answers that match the results obtained by the most expert assessors (indeed, that is the usual way their validity is assessed), and they appear to be particularly suitable in studies involving many investigators.

### 2.2.1 A taxonomy of PRO instruments

PRO instruments measure concepts ranging from the state of discrete symptoms or signs (e.g., pain severity or seizure frequency) to the overall state of a condition (e.g., depression, heart failure, angina, asthma, urinary incontinence, or rheumatoid arthritis), where both specific symptoms and the impact of the condition (e.g., on function, activities, or feelings) can be measured, to feelings about the condition or treatment (e.g., worry about getting worse, having to avoid certain situations, feeling different from others). PRO concepts can be general (e.g., improvement in physical function, psychological well-being, or treatment satisfaction) or specific (e.g., decreased frequency, severity, or how bothersome the symptoms are). PRO concepts can also be generic (i.e., applicable in a broad scope of diseases or conditions as in the case of physical functioning), condition-specific (e.g., asthma-specific), or treatment-specific (e.g., measures of the toxicities of a class of drugs such as interferons or opioids).

Some PRO instruments (e.g., health-related *quality of life *instruments) attempt to measure both the effectiveness and the side effects of treatment. PRO instruments that are used in clinical trials to support effectiveness claims should measure the adverse consequences of treatment separately from the effectiveness of treatment.

The specific attributes of a PRO instrument will affect the way it is developed, tested, and incorporated into a study protocol to support conclusions of treatment benefit. Table 1 lists some of the ways that PRO instruments can vary in their objectives, uses, and characteristics. When the FDA reviews a PRO instrument, our goal is to determine whether its characteristics are appropriate and adequate to support the study objectives.

**Table 1:** Taxonomy of PROs Used in Clinical Trials

**Attribute**	**Types**
Intended use of the measure	• To define entry criteria for study populations • To evaluate efficacy • To evaluate adverse events
Concepts measured	• Overall health status • Symptoms/signs, individually or as a syndrome associated with a medical condition • Functional status (physical, psychological or social) • Health perceptions (e.g., self-rating of health or worry about condition) • Satisfaction with treatment or preference for treatment • Adherence to medical treatment
Number of items	• Single item for single concept • Multiple items for single concept • Multiple items for multiple domains within a concept
Intended measurement population or condition	• Generic • Condition-specific • Population-specific
Mode of data collection	• Interviewer-administered • Self-administered, with or without supervision • Computer-administered or computer-assisted • Interactively administered (e.g., interactive voice response systems or Web-based systems)
Timing and frequency of administration	• As events occur • At regular intervals throughout a study • Baseline and end of treatment
Types of scores	• Single rating on a single concept (e.g., pain severity) • Index – single score combining multiple ratings of related domains or independent concepts • Profile – multiple uncombined scores of multiple-related domains • Battery – multiple uncombined scores of independent concepts • Composite – an index, profile, or battery
Weighting of items or concepts	• All items and domains are equally weighted • Items are assigned variable weights • Domains are assigned variable weights
Response options	• See Table 2 for examples of response options (types of PRO scales)

## 3. Evaluating pro instruments

The adequacy of a PRO instrument as a measure to support medical product claims depends on its developmental history and demonstrated measurement properties. Sponsors are encouraged to identify all endpoint measurement goals early in product development, before studies are initiated, to provide the basis for product approval or claim substantiation, allowing adequate time for PRO instrument identification, modification, or if necessary, new instrument development. A new PRO instrument can be developed or an existing instrument can be modified if sponsors determine that none is available, adequate, or applicable to their product development program. When considering an instrument that has been modified from the original, the FDA generally plans to evaluate the modified instrument just as it would a new one. Therefore, in such instances, we encourage sponsors to document the original development processes, all modifications made, and updated assessments of its measurement properties.

**Figure 1:**
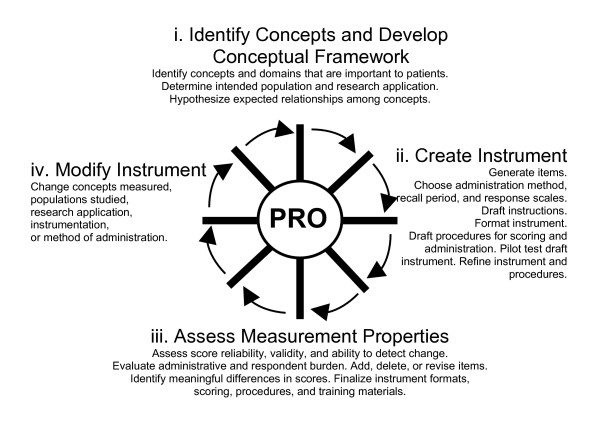
The PRO instrument development and modification process.

PRO instrument development, modification, and validation usually occur in a nonlinear fashion with a varying sequence of events, simultaneous processes, or iterations. This iterative process is presented as a *wheel and spokes *diagram, shown in Figure 1, and discussed in detail in Sections 3.1. – 3.4. One or more parts of the original process may be repeated in new PRO instrument development, modification, or change in application of an existing instrument. The following five sections describe the steps usually taken in instrument development.

### 3.1 Development of the conceptual framework and identification of the intended application

During the planning of clinical development programs, the FDA encourages sponsors to specify what claims they seek, determine what concepts underlie those claims, and then determine whether an adequate PRO instrument exists to assess and measure those concepts. If it doesn't, a new PRO instrument can be developed. The typical steps involved in the selection or development of PRO instruments for endpoints for clinical trials are described in the following sections.

#### 3.1.1 Identification of concepts and domains that are to be measured

One fundamental consideration in the development and use of a PRO instrument is whether the instrument's conceptual framework is appropriate and clearly defined. In some cases, of course, the question of what to measure may be obvious given the nature of the condition being treated. Generally, however, instrument developers choose the concepts and domains to be measured based on patient interviews along with reviews of the literature and expert opinion.

If documentation exists that a single item is a reliable and valid measure of the concept of interest (e.g., pain severity), a one-item PRO instrument may be a reasonable measure to support a claim concerning that concept. If the concept of interest is general (e.g., physical function), a single-item PRO instrument is usually unable to provide a complete understanding of the treatment's effect because a single item cannot capture all the domains of the general concept. For this reason, single-item questions about general concepts that imply multiple domains rarely provide sufficient evidence to support claims about that general concept. However, single-item questions about general concepts can be useful to help interpret multi-item measures of the same concept and to determine whether important items or domains of a general concept are missing (e.g., when results using single general questions do not correlate with results using a multi-item questionnaire, this may be evidence that the questionnaire is not capturing all the important domains of the concept contained in the claim). Evidence from the patient cognitive debriefing studies (i.e., the interview schedule, transcript, and listing of all concepts elicited by a single item) can be used to determine when a concept is adequately captured by a single item.

Multidomain PRO instruments can be used to support claims about a general concept if the PRO instrument has been appropriately developed and validated to measure the important and relevant domains of the general concept. The complex nature of multidomain PRO instruments, however, often raises significant questions about how to interpret and report results in a way that is not misleading. For example, if improvements in a score for a general concept (e.g., physical function) is driven by a single responsive domain (e.g., symptom improvement) while other important domains (e.g., physical abilities and activities of daily living) did not show a response, a general claim about improvements in physical function would not be supported. The FDA intends to review all evidence based on multidomain PRO measurements with particular attention to the precise claim that is supported by the results in the measured concepts or domains.

Documentation of the instrument development process should reveal the means by which the domains were identified and named. This helps substantiate the adequacy of the measure to support both the general concept and the named domains. If a sponsor desires to support a claim based on a portion of a multi-item instrument (a domain or an item), the development and validation process should ensure that the instrument supports the measurement of the claimed concept. For example, some broad health status measures include item lists of symptoms that are summed in an overall score. Individual items that contribute to the overall score (e.g., dyspnea) generally would not support a dyspnea claim unless the items were developed to measure the claimed concept (e.g., the items validly and reliably capture the impact of treatment on dyspnea).

**Figure 2:**
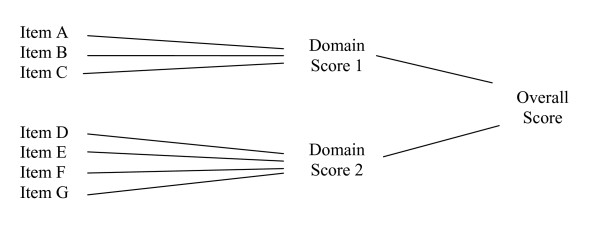
Diagram of a conceptual framework.

For measures of general concepts, the FDA intends to review how individual items are associated with each other, how items are associated with each domain, and how domains are associated with each other and the general concept of interest. A diagram of the expected relationships among the PRO items and domains can help reviewers evaluate these relationships. The diagram in Figure 2 depicts a generic example of a conceptual framework where Domain Score 1, Domain Score 2, and Overall Score each represent related but separate concepts. Items in this diagram are aggregated into domains. In some measures, domains can be aggregated into an overall score. These expectations should be specified before the validation process begins.

#### 3.1.2 Identification of the intended application of the PRO instrument

It is also important to consider whether the development and demonstrated measurement properties of a PRO instrument provide an adequate basis for its planned use in the study to support a claim. This is best established before the study commences, but would in any case be part of the FDA's application review. This is true whether the PRO instrument is generic, intended for use across multiple applications and populations, or specific, developed for a certain condition or population. The PRO instrument can be developed for a variety of roles, including defining trial entry criteria, including excessive severity, evaluating treatment benefit, or monitoring adverse events.

#### 3.1.3 Identification of the intended population

The FDA plans to compare the patient population used in the PRO instrument development process to the study populations enrolled in clinical trials to determine whether the instrument is appropriate to that population with respect to patient age, sex, ethnic identity, and cognitive ability. Specific measurement considerations posed by pediatric, cognitively impaired, or seriously ill patients are discussed in Section 3.5.

### 3.2 Creation of the PRO instrument

When developing a PRO instrument, sponsors are encouraged to assess its adequacy in the context of the following development processes.

#### 3.2.1 Generation of items

It is important to consider the procedures used to identify the set of items selected to measure a specific concept. PRO instrument items can be generated from literature reviews, transcripts from focus groups, or interviews with patients, clinicians, family members, researchers, or other sources. Depending on the conceptual framework, the FDA may review whether appropriate individuals and sources were used and how information gleaned from those sources was used in the PRO instrument development process.

PRO instrument item generation is incomplete without patient involvement. Item generation generally incorporates the input of a wide range of patients with the condition of interest to represent appropriate variations in severity and in population characteristics such as age or sex. The FDA plans to review instrument development (e.g., results from patient interviews or focus groups) to determine whether adequate numbers of patients have supported the opinion that the specific items in the instrument are adequate and appropriate to measure the concept.

Items that ask patients to respond hypothetically or that give patients the opportunity to respond on the basis of their desired condition rather than on their actual condition are not recommended. For example, in assessing the concept *performance of daily activities*, it is more appropriate to ask whether or not the respondent performs specific activities (and if so, with how much difficulty) than whether or not he or she can perform daily activities (because patients may report they are able to perform a task even when they never do so). Of course, it would be critical to know that each item refers to something that patients actually do.

It is also important to consider all of the item generation techniques used, including any theoretical approach used, the populations studied, sources of items, selection and reduction of items, cognitive debriefing interviews, pilot testing, importance ratings, and quantitative techniques for item evaluation such as factor analysis and item-response analysis.

#### 3.2.2 Choice of the data collection method

Sponsors should consider the method of data collection and all procedures and protocols associated with instrument administration, including instructions to interviewers, instructions for self-administration, instructions for supervising self-administration, case report forms or examples of electronic PRO instruments, and other special considerations specific to the mode of administration including data quality control procedures. Modes of administration include interview, paper-based, electronic, Web-based, and interactive voice response formats. The FDA intends to review the comparability of data obtained when using multiple modes of administration to determine whether pooling of results from the multiple modes is appropriate.

#### 3.2.3 Choice of the recall period

Sponsors should also evaluate the rationale and the appropriateness of the recall period for a PRO instrument. To this end, it is important to consider patients' ability to accurately recall the information requested as proposed. The choice of recall period that is most suitable depends on the purpose and intended use of the instrument, the characteristics of the disease/condition, and the treatment to be tested. When evaluating PRO-based claims, the FDA intends to review the study protocol to determine what steps were taken to ensure that patients understand the appropriate recall period. If a patient diary or some other form of unsupervised data entry is used, the FDA plans to review the protocol to determine what measures are taken to ensure that patients make entries according to the study design and not, for example, just before a clinic visit when their reports will be collected.

PRO instruments that require patients to rely on memory, especially if they must recall over a period of time, or to average their response over a period of time may threaten the accuracy of the PRO data. It is usually better to construct items that ask patients to describe their current state than to ask them to compare their current state with an earlier period or to attempt to average their experiences over a period of time.

#### 3.2.4 Choice of response options

It is also important to consider whether the response options are consistent with the purpose and intended use of the PRO instrument. Table 2 describes the types of response options that are typically used in clinical trials.

**Table 2:** Types of Response Options

**Type**	**Description**
Visual analog scale (VAS)	A line of fixed length (usually 100 mm) with words that anchor the scale at the extreme ends and no words describing intermediate positions. Patients are instructed to place a mark on the line corresponding to their perceived state. These scales often produce a false sense of precision.
Anchored or categorized VAS	A VAS that has the addition of one or more intermediate marks positioned along the line with reference terms assigned to each mark to help patients identify the locations (e.g., half-way) between the ends of the scale.
Likert scale	An ordered set of discrete terms or statements from which patients are asked to choose the response that best describes their state or experience.
Rating scale	A set of numerical categories from which patients are asked to choose the category that best describes their state or experience. The ends of rating scales are anchored with words but the categories do not have labels.
Event log	Specific events are recorded as they occur using a patient diary or other reporting system (e.g., interactive voice response system)
Pictorial scale	A set of pictures applied to any of the other types of response options. Pictorial scales are often used in pediatric questionnaires but also have been used for patients with cognitive impairments and for patients who are otherwise unable to speak or write.
Checklist	Checklists provide a simple choice between a limited set of options, such as *Yes*, *No*, and *Don't know*. Some checklists ask patients to place a mark in a space if the statement in the item is true. Checklists are reviewed for completeness and nonredundancy.

Response choices are generally considered appropriate when:

• Wording used in responses is clear and appropriate (e.g., anchoring a scale using the term *normal *assumes that patients understand what is normal).

• Responses are appropriate for the intended population. For example, patients with visual impairment may find the VAS difficult to complete.

• Responses offer a clear distinction between choices (e.g., patients may not distinguish between *intense *and *severe *if both are offered as response choices to describe their pain).

• Instructions to patients for completing the questionnaire and selecting response options are adequate.

• The number of response options is justified.

• Response options are appropriately ordered and appear to represent equal intervals.

• Response options avoid potential ceiling or floor effects (e.g., introducing more categories to capture worsening or improvement so that fewer patients respond at the top or bottom of the response continuum).

• Response options do not bias the direction of responses (e.g., offering one negative choice, one neutral choice, and two or more positive choices on a scale makes it more likely for patients to respond that they feel or function better).

#### 3.2.5 Evaluation of patient understanding

Sponsors are encouraged to examine the procedures used with patients to determine readability and understanding of the items included in the PRO instrument. The FDA's evaluation of these procedures is likely to include a review of a cognitive debriefing report containing the readability test used, the script used in patient cognitive debriefing interviews, the transcript of the interviews, the analysis of the interview results, and the actions taken to delete or modify an item in response to the cognitive debriefing interview or pilot test results.

#### 3.2.6 Development of format, instructions, and training

PRO study results can vary according to the instructions to patients or the training given to the interviewer or persons supervising PRO data collection. Sponsors should consider all PRO instrument instructions and procedures contained in publications and user manuals provided by developers, including procedures for reviewing completed questionnaires and re-administration to avoid missing data or clarify responses. Other important considerations include the format of the questionnaire, the final wording of PRO instruments as implemented in clinical trials, and any potentially important changes in presentation or format. Examples of changes that can alter the way that patients respond to the same set of questions include:

• Changing an instrument from paper to electronic format

• Changing the timing of or procedures for PRO instrument administration within the clinic visit

• Changing the order of items or deleting portions of a questionnaire

• Changing the instructions or the placement of instructions within the PRO instrument

It is important that the PRO instrument format used in the clinical trial be consistent with the format that is used in the instrument validation process. *Format *refers to the exact appearance of the instrument. Instrument format is specific to the mode of administration, including paper and pencil, interviewer-administered or supervised, or electronic data collection. The FDA plans to review the PRO instrument in the format used in the clinical trial case report forms, including the order and numbering of items, the presentation of response options in single response or grid formats, the grouping of items, patterns for skipping questions that are not applicable, and all instructions to patients in the interview schedule or on the questionnaire.

The FDA recommends that the PRO instrument development process includes the generation of a user manual that specifies how to incorporate the instrument into a clinical trial in a way that minimizes administrator burden, patient burden, missing data, and poor data quality.

#### 3.2.7 Identification of preliminary scoring of items and domains

For each item, numerical scores are generally assigned to each answer category based on the most appropriate scale of measurement for the item (e.g., nominal, ordinal, interval, or ratio scales). The FDA intends to consider whether a PRO measure conforms to assumptions that the response choices represent appropriate intervals by reviewing distributions of item responses.

A scoring algorithm creates a single score from multiple items. Equally weighted scores for each item are appropriate only when the responses to the items are relatively uncorrelated. Otherwise, the assignment of equal weights will overweight correlated items and underweight independent items. Even when items are uncorrelated, assigning equal weights to each item may overweight certain items if the number of response options or the values associated with response options varies by item. The same weighting concerns apply with added complexity when combining domain scores into a single overall score.

When empirically determined patient preference ratings are used to weight items or domains, the FDA also intends to review the composition of samples and the process used to determine the preference weights. Because preference weights are often developed for use in resource allocation (e.g., as in cost-effectiveness analysis that may use predetermined community weights), it is tempting to use those same weights in the clinical trial setting to demonstrate treatment benefit. However, this practice is discouraged unless the relationship of the preference weights to the intended study population is known and found adequate and appropriate.

#### 3.2.8 Assessment of respondent and administrator burden

Undue physical, emotional, or cognitive strain on patients are burdens that will generally decrease the quality and quantity of PRO data. Factors that can contribute to respondent burden include the following:

• Length of questionnaire or interview

• Formatting

• Font size too small to read easily

• New instructions for each item

• Words or sentence structures that require a technical knowledge or developmental level beyond that of the patients in the trials

• Requirement that patients consult records to complete responses

• Privacy of the setting in which the PRO is completed (e.g., not providing a private space for patients to complete questionnaires containing sensitive information about their sexual performance or substance abuse history)

• Inadequate time to complete questionnaires or interviews

• Literacy level too high for population

• Questions that patients are unwilling to answer

• Perception by patients that the interviewer wants or expects a particular response

The degree of respondent burden that is acceptable for instruments in clinical trials depends on the frequency and timing of PRO assessments in a protocol and on the severity of the illness or toxicity of the treatment studied. For example, if the questionnaire contains instructions to skip one or more questions based on responses to a previous question, respondents may fail to understand what is required and make errors in responding or find the assessment too complicated to complete. Sponsors should consider missing data and the refusal rate as possible indications of unacceptable patient burden or inappropriate items or response options.

#### 3.2.9 Confirmation of the conceptual framework and finalization of the instrument

The FDA intends to examine the final version of an instrument in light of its development history, including documentation of the complete list of items generated and the reasons for deleting or modifying items, as illustrated in Table 3. It will be important to determine from empirical data submitted whether the conceptual framework (e.g., the expected relationships between items, domains, and measurement concepts as diagrammed in Figure 2) have been demonstrated.

**Table 3:** Common Reasons for Changing PRO Instruments During Initial Development

**Item Property**	**Reason for Change or Deletion**
Clarity or relevance	• Reported as not relevant by a large segment of the population of interest • Generates an unacceptably large amount of missing data points • Generates many questions or requests for clarification from patients as they complete the PRO instrument • Patients interpret items and responses in a way that is inconsistent with the conceptual framework
Response range	• A high percent of patients respond at the floor (worst end of the response scale) or ceiling (optimal end of the response scale) • Patients note that none of the response choices apply to them • Item means are highly skewed
Variability	• All patients give the same answer (i.e., no variance) • Most patients choose only one of the response choices • Differences among patients are not detected when important differences are known
Reproducibility	• Unstable scores over time when there is no logical reason for variation from one assessment to the next
Inter-item correlation	• Item uncorrelated with other items in the same concept of interest
Ability to detect change	• Item is nonresponsive (i.e., does not change when there is a known change in the concepts of interest)
Item discrimination	• Item is highly correlated with measures of concepts other than the one it is intended to measure
Redundancy	• Item duplicates information collected with other items that have equal or better measurement properties

### 3.3 Assessment of measurement properties

The FDA generally intends to review a PRO instrument for: reliability, validity, ability to detect change, and interpretability (e.g., minimum important difference). The FDA plans to review the measurement properties that are specific to the documented conceptual framework, confirmed scoring algorithm, administration procedures, and questionnaire format in light of the study population, study design, and statistical analysis plan. The sociodemographic and medical characteristics of any sample used to develop or validate a PRO instrument determine its appropriateness for future clinical study settings. (See Table 4.)

**Table 4:** Measurement Properties Reviewed for PRO Instruments Used in Clinical Trials

**Measurement Property**	**Test**	**What is Assessed**	**FDA Review Considerations**
Reliability	Test-retest	Stability of scores over time when no change has occurred in the concept of interest	Does the PRO instrument reliably measure the concepts it was designed to measure?
	Internal consistency	Whether the items in a domain are intercorrelated, as evidenced by an internal consistency statistic (e.g., coefficient alpha)	Were appropriate reliability tests conducted?
	Inter-interviewer reproducibility (for interviewer-administered PROs only)	Agreement between responses when the PRO is administered by two or more different interviewers	What was the quality of the evidence of reliability?
Validity	Content-related	Whether items and response options are relevant and are comprehensive measures of the domain or concept	Do items in the verbatim copy of the PRO instrument appear to measure the concepts they are intended to measure in a useful way?
			Have patients similar to those participating in the clinical trial confirmed the completeness and relevance of all items?
	Ability to measure the concept (also known as construct-related validity; can include tests for discriminant, convergent, and known-groups validity)	Whether relationships among items, domains, and concepts conform to what is predicted by the conceptual framework for the PRO instrument itself and its validation hypotheses.	Do observed relationships between the items and domains confirm the hypotheses in the conceptual framework? Do results compare favorably with results from a similar but independent measure?
			Do results distinguish one group from another based on a prespecified variable that is relevant to the concept of interest?
	Ability to predict future outcomes (also known as predictive validity)	Whether future events or status can be predicted by changes in the PRO scores	Do PRO scores predict subsequent events or outcomes accurately?
Ability to detect change	Includes calculations of effect size and standard error of measurement among others	Whether PRO scores are stable when there is no change in the patient, and the scores change in the predicted direction when there has been a notable change in the patient as evidenced by some effect size statistic. Ability to detect change is always specific to a time interval.	Has ability to detect change been demonstrated in a comparative trial setting, comparing mean group scores or proportion of patients who experienced a response to the treatment?
			Has ability to detect change been assessed for the time interval appropriate to study?
Interpretability	Smallest difference that is considered clinically important; this can be a specified difference (the minimum important difference (MID)) or, in some cases, any detectable difference. The MID is used as a benchmark to interpret mean score differences between treatment arms in a clinical trial	Difference in mean score between treatment groups that provides convincing evidence of a treatment benefit. Can be based on experience with the measure using a distribution-based approach, a clinical or nonclinical anchor, an empirical rule, or a combination of approaches. The definition of an MID using a clinical anchor is sometimes called an MCID.	The FDA is specifically requesting comment on appropriate review of derivation and application of an MID in the clinical trial setting.
	Responder definition – used to identify responders in clinical trials for analyzing differences in the proportion of responders between treatment arms	Change in score that would be clear evidence that an individual patient experienced a treatment benefit. Can be based on experience with the measure using a distribution-based approach, a clinical or nonclinical anchor, an empirical rule, or a combination of approaches.	The FDA is specifically requesting comment on appropriate review of derivation and application of responder definitions when used in clinical trials.

#### 3.3.1 Evaluation of reliability

Because clinical trials involve change over time, the adequacy of a PRO instrument for use in a clinical trial depends on its reliability. Because clinical trials are intended to provide unbiased estimates of true treatment impact, systematic and/or other changes in measurement methods may undermine the purpose of the trial.

Test-retest reliability is the most important type of reliability for PRO instruments used in clinical trials. Test-retest is most informative when the time interval chosen between the test and retest is appropriate for identifying stability in reference to the clinical trial protocol.

Internal consistency reliability, in the absence of test-retest reliability, does not generally constitute sufficient evidence of reliability for clinical trial purposes. When PRO instruments are interviewer-administered, inter-interviewer reproducibility is critical.

#### 3.3.2 Evaluation of validity

The FDA recognizes that the validation of an instrument is an ongoing process and that validity relates to both the instrument itself and how it is used. Sponsors should consider a PRO endpoint for evidence of content-related validity, the instrument's ability to measure the stated concepts, and the instrument's ability to predict future outcomes, as illustrated in Table 4.

If instrument developers expected the instrument to give results for the measured concept similar to those measured by existing PRO or non-PRO measures (e.g., physical or physician-based measures), the FDA is interested in documented demonstration of those relationships to determine whether the instrument convincingly measures that concept and can therefore support a claim about that concept. If developers expected the instrument to discriminate between patient groups (e.g., between patients with different levels of severity), the FDA is interested in evidence that shows the instrument meaningfully discriminates.

In some cases, some types of validity testing are not possible due to the nature of the concept to be measured. In such instances, the FDA generally plans to review the cumulative evidence for the appropriate use of the measure and apply it to the interpretation of clinical study results.

#### 3.3.3 Evaluation of ability to detect change

When a concept is expected to change, the values for the PRO instrument measuring that concept should change. If there is clear evidence that patient experience relative to the concept has changed, but the PRO scores do not change, the validity of the PRO instrument should be questioned. If there is evidence that PRO scores are affected by changes that are not specific to the concept of interest, the validity of the PRO instrument should be questioned.

The ability of an instrument to detect change influences the sample size needed to evaluate the effectiveness of treatment. The extent to which the PRO instrument's ability to detect change varies by important patient subgroups (e.g., sex, race, age, or ethnicity) can affect clinical trial results. It is important to identify any important subgroup differences in ability to detect change so that these differences can be taken into account in assessing results.

#### 3.3.4 Choice of methods for interpretation

The following sections describe some of the methods that have helped sponsors and the FDA interpret clinical trial results based on PRO endpoints.

##### 3.3.4.1 Defining a minimum important difference

Many PRO instruments are able to detect mean changes that are very small; accordingly it is important to consider whether such changes are meaningful. Therefore, it is appropriate for a critical distinction to be made between the mean effect seen (and what effect might be considered important) and a change in an individual that would be considered important, perhaps leading to a definition of a *responder*. For many widely used measures (pain, treadmill distance, HamD), the ability to show *any *difference between treatment groups has been considered evidence of a relevant treatment effect. If PRO instruments are to be considered more sensitive than past measures, it can be useful to specify a minimum important difference (MID) as a benchmark for interpreting mean differences. An MID is usually specific to the population under study.

The FDA has reviewed MIDs derived in many ways. Examples include:

• Mapping changes in PRO scores to clinically relevant and important changes in non-PRO measures of treatment outcome in the condition of interest (e.g., when PRO measures of asthma or COPD are mapped to spirometry scores).

• Mapping changes in PRO scores to other PRO scores to arrive at an MID that is appreciable to patients (e.g., when multi-item PROs are mapped to a single question asking the patient to rate his or her global impression of change since the start of treatment). A problem with this approach is that it uses individual rates to reach a conclusion about mean effects. It may be more useful to look at the distribution of individual effects in treatment and control groups.

• Using a distribution-based approach (e.g., defining the MID as 0.5 times the standard deviation). This, of course, may bear no relation to the patient's assessment and is usually inadequate in isolation.

• Using an empirical rule (e.g., 8 percent of the theoretical range of scores). Again, this arbitrary approach does not take into account patient preferences or assessment.

If an MID is to be applied to clinical study results, it is generally helpful to use a variety of methods to discover whether concordance among methods confirms the choice of an MID (see appendix point 3).

##### 3.3.4.2 Definition of responders

There may be situations where it is more reasonable to characterize the meaningfulness of an individual's response to treatment than a group's response, and there may be interest in characterizing an individual patient as a responder to treatment, based upon prespecified criteria backed by empirically derived evidence supporting the responder definition as a measure of benefit. Such examples include categorizing a patient as a responder based upon a prespecified change from baseline on one or more scales; a change in score of a certain size or greater (e.g., a 2-point change on an 8-point scale); or a percent change from baseline (see appendix point 4).

### 3.4 Modification of an existing instrument

When a PRO instrument is modified, additional validation studies may be needed to confirm the adequacy of the modified instrument's measurement properties. The extent of additional validation recommended depends on the type of modification made. For example, small nonrandomized studies may be adequate to assess the results of changing a response scale from vertical to horizontal. On the other hand, if the PRO instrument is to be used in an entirely new population of patients, a small randomized study to ascertain the measurement properties in the new population may minimize the risk that the instrument will not perform adequately in a phase 3 study.

The FDA intends to consider a modified instrument as a different instrument from the original and will consider measurement properties to be version-specific. The FDA recommends additional validation to support the development of a modified PRO instrument when one or more of the following modifications occur.

#### 3.4.1 Revised measurement concept

An instrument that is developed and validated to measure one concept is used to measure a different concept. For example:

• A single domain from a multiple domain PRO is administered without the other domains

• Response options are changed to assess a different quality (e.g., frequency versus how bothersome)

• An index or composite score is used to summarize multiple PRO concepts/domains when existing validation applies only to concept/domain-specific scores

• Items from an existing PRO instrument are used to create a new instrument

• One or more items from an existing instrument are used to support a claim for a concept the items were not developed to measure

#### 3.4.2 Application to a new population or condition

An instrument developed for use in one population or condition is used in a different patient population or condition. For example:

• Patients in the proposed trial have a disease, condition, or severity level that is different from that of the patient population used for instrument development and validation

• Patients in the proposed trial differ in age, gender, race, or developmental or life stage from those for instrument development and validation

#### 3.4.3 Changed item content or instrument format

An instrument is altered in item content or format. This includes changes in the following:

• Number of items (more or fewer) used to assess a concept or domain

• Wording or placement of instructions

• Wording or order of the items

• Wording, scaling, ordering, or number of response options

• Recall period associated with an item

• Point of reference for comparison for an item or domain

• Weighting of items

• Scoring (including creation of summary scores, subdomain scores, or cut-points)

• Any changes that could alter the patient's interpretation of the instructions, items, or response options

#### 3.4.4 Changed mode of administration

An instrument's data collection mode is altered. For example:

• An interviewer-administered or supervised questionnaire is modified for self-administration (skip patterns can be a problem in this situation)

• Paper-and-pencil self-administered PRO is modified to be administered by computer or other electronic device (e.g., computer adaptive testing, interactive voice response systems, Web-based questionnaire administration, computer)

• Instructions or procedures for administration within a trial differ from those used in validation studies (can alter the meaning of the responses from that of the original version)

#### 3.4.5 Changed culture or language of application

An instrument developed in one language or culture is adapted or translated for use in another language or culture. The FDA recommends that sponsors provide evidence that the methods and results of the translation process were adequate to ensure that the validity of the responses is not affected. Some examples include the following:

• PRO instruments are developed initially in one language, culture, or ethnic group and are used subsequently in another

• PRO instruments developed and validated outside the United States are applied to the U.S. population

Sponsors should consider whether generally accepted standards for translation and cultural adaptation have been used to support the validity of data from a translated/adapted PRO instrument, including but not restricted to the following:

• The background and experience of the persons involved in the translation/adaptation

• The translation/adaptation methodology used

• The harmonization of different versions

• The evidence that measurement properties for translated versions are comparable

#### 3.4.6 Other changes

Other changes to the PRO instrument or the way in which it is assessed that may necessitate additional validation include:

• The PRO instrument was not developed and validated for use in a clinical trial

• A PRO instrument developed and previously used as a stand-alone assessment is included as a part of a battery of measures

• A PRO developed to measure a treatment benefit is subsequently used to measure a decrement as interpreted by a score change in the opposite direction

### 3.5 Development of PRO instruments for specific populations

Measurement of PRO concepts in children and youth, and in patients who have cognitive impairment, introduces challenges in addition to those already mentioned. These are discussed in the following sections.

#### 3.5.1 Children and youth

In general, the review issues related to the development and validation of pediatric PRO instruments are similar to those detailed for adults. It is important that PRO instruments developed for adults are not used in pediatric populations unless the measurement properties are similar in all age groups tested. We recommend that instruments intended for use in pediatric populations be rigorously developed and validated according to the principles described earlier. Additional review issues for PRO instruments applied in children and youth include age-related vocabulary, language comprehension, comprehension of the health concept measured, and duration of recall. Instrument development and validation testing within fairly narrow age groupings is important to account for developmental differences and to determine the lower age limit at which children can understand the questions and provide reliable and valid responses that can be compared across age categories.

#### 3.5.2 Patients cognitively impaired or unable to communicate

Over the course of some clinical trials, it can be anticipated that patients may become too ill to complete a questionnaire or to respond to an interviewer. In such cases, proxy reporting may help to prevent missing data. When this situation is anticipated, the FDA encourages the inclusion of proxy reports in parallel with patient self-report from the beginning of the study (i.e., even before the patient is no longer able to answer independently) so that the relationship between the patient reports and the proxy reports can be assessed.

## 4. Study design

The same study design principles that apply to other endpoint measures apply to PROs. This section, therefore, focuses primarily on issues unique to PROs.

### 4.1 General protocol considerations

If the goal of PRO measurement is to support claims, we recommend that measurement of the PRO concept be clearly stated as a specific study objective. It is important that the protocol include the exact format and version of the specific PRO instrument to be administered. In the process of considering the NDA/BLA/PMA or NDA/BLA/PMA supplement, the FDA intends to compare both the planned and actual use of the PRO instrument and its analysis.

#### 4.1.1 Blinding and randomization

Because responses to PRO measures are subjective, representing a patient's impression, open-label studies, where patients and investigators are aware of assigned therapy, are rarely credible. Patients who know they are in an active treatment group may overestimate benefit while those who know they are not receiving active treatment may underreport any improvement actually experienced. Every effort should be made to assure that patients are masked to treatment assignment throughout the trial. If the treatment has obvious effects, blinding may be difficult. The impact of possible unblinding is important to consider in the interpretation of study results.

The importance of blinding can be determined, in part, by the characteristics of the PRO instrument used. For example, questions that ask how patients' current status compares to baseline seem likely to be more influenced by unblinding (optimism can readily be expressed as a favorable comparison) than questions that ask about current status (which requires a current assessment, not a statement about duration). Questions that ask for current status, or PRO instruments that ask many questions, are harder to answer in a biased way when previous answers are not available. For the same reasons, allowing patients access to previous responses can bias results when unblinding is a possibility. This is, however, an area that could benefit from rigorous study.

There are certain situations, particularly in the development of medical devices, where blinding is not feasible and other situations where there is no reasonable control group (and therefore no randomization). When a PRO instrument appears useful in assessing patient benefit in those situations, the FDA encourages sponsors to confer with the appropriate review division.

#### 4.1.2 Clinical trial quality control

Study quality can be optimized at the design stage by specifying procedures to minimize inconsistencies in trial conduct. Examples of standardized instructions and processes that may appear in the protocol include:

• Standardized training and instructions to patients for self-administered PRO instruments

• Standardized interviewer training and interview format for PRO instruments administered in an interview format

• Standardized instructions for the clinical investigators regarding patient supervision, timing and order of questionnaire administration during or outside the office visit, processes and rules for questionnaire review for completeness, and documentation of how and when data are filed, stored, and transmitted to or from the study site

#### 4.1.3 Designing the trial to avoid data missing due to withdrawal from exposure

Sometimes patients fail to report for visits, fail to complete questionnaires that contain response endpoints, or withdraw from assigned treatment prior to planned completion of a clinical trial without contributing PRO information. The resulting missing data can introduce bias and interfere with the ability to compare effects in the test group with the control group because only a subset of the initial randomized population contributes, and these patient groups may no longer be comparable. Missing data is a major challenge to the success and interpretation of any clinical trial.

The protocol can increase the likelihood that a trial will still be informative by establishing plans for gathering all treatment-related reasons for patients withdrawing from a trial and by trying to minimize patient dropouts prior to trial completion. We recommend the study protocol describe how missing data will be handled in the analysis. It could also establish a process by which PRO measurement is ascertained before or shortly after patient withdrawal from treatment exposure due to lack of efficacy or toxicity.

### 4.2 Frequency of measurements

The frequency of PRO assessment depends on the natural history of the disease and the nature of the treatment. Some diseases, conditions, or study designs may necessitate more than one baseline assessment and several PRO assessments during treatment. The frequency of PRO assessment should correspond with the demonstrated measurement properties of the instrument and with the planned data analysis.

### 4.3 Duration of study

It is also important to consider whether the duration of the study is of adequate length to support the proposed claim and assess a durable outcome in the disease or condition being studied. Generally, duration of follow-up with a PRO assessment should be at least as long as for other measures of effectiveness. It should be noted, however, that the study duration appropriate for the PRO-related study objective may not be the same as the study duration for other study endpoints. In a trial for a progressive disease where the PRO concept of interest does not change until after the follow-up required for other clinical efficacy parameters, longer study duration can be indicated.

### 4.4 Design considerations for multiple endpoints

The hierarchy of endpoints is determined by the stated objectives of the trial and the clinical relevance and importance of each specific measure independently and in relationship to each other. A PRO instrument could be the primary endpoint measure of the study, a co-primary endpoint measure in conjunction with other objective or physician-rated measurements, or a secondary endpoint measure whose analysis would be considered according to a hierarchical sequence. The FDA recommends that the study protocol define the study endpoint measures and the criteria for the statistical analysis and interpretation of results, including a clear specification of the conditions for a positive study conclusion.

### 4.5 Planning for study interpretation

The FDA recommends that sponsors discuss with the appropriate review division how best to plan for the interpretation of study findings. In some cases, the FDA may request an *a priori *definition of the minimum observed difference between treatment group means (i.e., MID) that will serve as a benchmark to interpret whether study findings are conclusive. In other cases, the FDA may request an *a priori *definition of a treatment responder that can be applied to individual patient changes over time. Prespecification of methods for interpretation is particularly important with new or unfamiliar instruments or when patient dropouts, withdrawals from exposure, or missing data are expected (e.g., in studies where repeated PRO measurement is planned). See Section 5.5 for guidance on interpretation considerations for a study's statistical analysis plan.

### 4.6 Specific concerns when using electronic PRO instruments

When electronic PRO instruments are used, sponsors should plan carefully to ensure that FDA regulatory requirements are met for sponsor and investigator record keeping, maintenance, and access (see appendix point 5). These responsibilities are independent of the method used to record clinical trial data and, therefore, apply to electronic PRO data. Sponsors are responsible for providing investigators with the information they need to conduct the investigation properly, for monitoring the investigation, for ensuring that the investigation is conducted in accordance with the investigational plan, and for permitting the FDA to access, copy, and verify records and reports relating to the investigation.

The principal record keeping requirements for clinical investigators include the preparation and maintenance of adequate and accurate case histories (including the case report forms and supporting data), record retention, and provision for the FDA to access, copy, and verify records (i.e., source data verification). The investigator's responsibility to control, access, and maintain source documentation can be satisfied easily when paper PRO instruments are used, because the subject usually returns the diary to the investigator who either retains the original or a certified copy as part of the case history. The use of electronic PRO instruments, however, may pose a problem if direct control over source data is maintained by the sponsor or the contract research organization and not by the clinical investigator. The FDA considers the investigator to have met his or her responsibility when the investigator retains the ability to control and provide access to the records that serve as the electronic source documentation for the purpose of an FDA inspection. The FDA recommends that the study protocol, or a separate document, clearly specify how the electronic PRO source data will be maintained.

In addition, the FDA has previously provided guidance to address the use of computerized systems to create, modify, maintain, archive, retrieve, or transmit clinical data to the agency (see appendix point 6) and to clarify the requirements and application of 21 CFR part 11 (see appendix point 7). Because electronic PRO data (including data gathered by personal digital assistants or phone-based interactive voice recording systems) are part of the case history, the FDA expects electronic PRO data to be consistent with the data standards described in that guidance. Sponsors should plan carefully to establish appropriate system and security controls, as well as cybersecurity and system maintenance plans that address how to ensure data integrity during network attacks and software updates.

Sponsors should also plan to avoid the following (see appendix point 8)

• Direct PRO data transmission from the PRO data collection device to the sponsor (i.e., the sponsor should not have exclusive control of the source document)

• The existence of only one database without backup (i.e., risk of data corruption or loss during the trial with no way to reconstitute or verify the data)

• Removal of investigator accountability for confirming the accuracy of the data

• Loss of adverse event data

• Access to unblinded data

• Inability of an FDA investigator to inspect, verify, and copy the data at the clinical site during an inspection

• An insecure system that allows for easily alterable records.

## 5. Data analysis

Incorporating PRO instruments as study endpoint measures introduces challenges in the analysis of clinical trial data. Some of these challenges are discussed in the following sections.

### 5.1 General statistical considerations

The statistical analysis considerations for PRO endpoints are not unlike statistical considerations for any other endpoint used in drug development (see appendix point 9). We recommend that the principal features of the planned statistical analysis of the data be described in the statistical section of the protocol and in a detailed elaboration of the analysis often called the Statistical Analysis Plan (SAP). The FDA intends to determine the adequacy of study data to support claims in light of the prespecified method for endpoint analysis. Unplanned or post hoc statistical analyses are usually viewed as exploratory and, therefore, unable to serve as the basis of a claim of effectiveness.

### 5.2 Statistical considerations for using multiple endpoints

It is important that the study protocol specify all endpoints that will be considered, including each domain score targeted to support a specific claim. The SAP should describe the planned primary analysis in detail, noting whether the endpoint will be analyzed as a continuous variable (mean scores), dichotomous variable (success/failure), or some graded response, the primary and secondary endpoints, corrections for multiplicity, and the specific statistical methods planned.

In some situations, the SAP can specify that two or more variables must be statistically demonstrated to be superior to control group findings to support a claim. This may be the case, for example, when a clinician-reported endpoint and a patient-reported endpoint both need to be shown better than the control. Control for multiplicity (i.e., adjustment of the Type I error) generally is not a concern when all endpoints are shown to be superior to those of the comparison group, but we recommend carefully considering the impact of choosing multiple primary endpoints on Type II error and sample size. The sample size of the trial may be affected by how many endpoints are measured, the overall strategy planned to integrate all endpoints in the SAP, and the decision rule for declaring a successful study outcome.

Because each PRO item or domain often can represent an endpoint that could imply a distinct claim on its own, we recommend careful planning to avoid substantial increases in Type 1 error from multiple endpoints. If it is important in a study to demonstrate that PROs have the same directional effect as other measures of treatment benefit, then statistical procedures can be considered to minimize the impact of multiple endpoint comparisons.

There is no single best statistical procedure for multiplicity adjustment because the choice of procedure depends upon the study objectives, the most important endpoints among the collection, and other considerations. Some of the statistical procedures that can be useful for a more efficient analysis approach include methods that prespecify a sequence or order of the testing or that have a hierarchy of comparisons that first need to be satisfied before others are considered for testing (i.e., closed testing procedures, gatekeeper strategies). Generally, these statistical methods are less conservative than the classical Bonferroni or other statistical multiplicity adjustments that are used to control false positive conclusions from a family of eligible hypotheses. Another reason to consider less conservative methods is to adjust for what are often strong correlations among the endpoints (causing a Bonferroni adjustment to be too conservative). These strategies reduce the need for more stringent statistical tests for the subsequent endpoints, but do not allow statistical testing for endpoint combinations not prespecified.

A multidomain PRO measure can successfully support a claim based on one or a subset of the domains measured if an *a priori *analysis plan prespecifies the domains that will be targeted as endpoints for the study. However, demonstration that only a subset of domains is affected by treatment (e.g., the physical function domain) generally will not support a general claim (e.g., a claim of *improved HRQL*) because such a claim implies improvement on all domains that are important to the general concept. Use of domain subsets as study endpoints presupposes that the PRO instrument was adequately developed and validated to measure the subset of domains independently from the other domains.

The FDA recommends that the sponsor discuss with the FDA in advance of the study the appropriateness of the statistical strategies proposed in the SAP.

### 5.3 Statistical considerations for composite measures

Understanding the usefulness and measurement properties of a composite endpoint (i.e., an index, profile, or battery of scores) is an iterative process that evolves over time. Rules for interpretation of composite measures depend on substantial clinical experience with the measure in the clinical trial setting. Development of a composite endpoint at the time the confirmatory clinical study protocol is generated is discouraged unless there is substantial prior empirical evidence of the value of the chosen components of the composite. Though one reason for use of a composite is to reduce the multiplicity problems associated with multiple separate endpoints, composites can do so only if it is agreed that treatment impact on each of the endpoints is of value and if the endpoints move in the same direction.

Establishing benefit is difficult if only one component of a composite endpoint responds to the treatment. For example, a treatment may relieve certain symptoms or improve functioning but this benefit may not be detected using a composite score that includes other endpoints (e.g., psychological or emotional well-being) that fail to improve with the treatment. In any such composite, it is critical to ensure that patients enrolled in a clinical study are impaired in all domains (e.g., psychological or emotional well-being) because they cannot improve in domains if they are not impaired in whatever concept the domain measures.

Multiplicity problems arise when the multiple individual components of a composite endpoint are intended as possible claims. In general, individual components of a composite measure will not be adequate to support a claim unless the components are prespecified in the SAP as separate endpoints, either sharing overall study alpha (co-primary endpoints) or identified in a sequential analysis, and the study results are found statistically and clinically meaningful in the context of the total composite and other individual component results.

In general, if analysis of scores for the individual component endpoints of a composite shows the improvement is driven primarily by a single domain (e.g., performance of a specific activity), the findings for the composite score would not support a general claim (e.g., psychological or emotional benefit, or even general physical state if all that is shown is symptom improvement).

### 5.4 Statistical considerations for patient-level missing data

The FDA recommends that the SAP address plans for how the statistical analyses will handle missing data when evaluating treatment efficacy and when considering patient success or patient response.

#### 5.4.1 Missing items within domains

At a specific patient visit, a domain measurement may be missing some, but not all, items. Defining rules that specify the number of items that can be missing and still consider the domain to have been measured is one approach to handling this type of missing data. Rules for handling missing data should be specific to each PRO instrument and should usually be determined during the instrument development and validation process. The FDA recommends that all rules be specified in the SAP. For example, the SAP can specify that a domain will be treated as missing if more than 25 percent of the items are missing; if less than 25 percent of the items are missing, the domain score can be taken to be the average of the nonmissing items.

#### 5.4.2 Missing entire domains or entire measurements

When the amount of missing data becomes large, study results can be inconclusive. As described earlier, the FDA encourages prespecified procedures in the study protocol, particularly when patients discontinue study treatment. Because missing data may be due to the treatment received or the underlying disease and can introduce bias in the analysis of treatment differences and conclusions about treatment impact, the FDA encourages sponsors to obtain data on each patient at the time of withdrawal to determine the reason for withdrawal. When available, this information can be taken into account in the analysis.

A variety of statistical strategies have been proposed in the literature and applications to the FDA to deal with missing data due to patient withdrawal from assigned treatment exposure prior to planned completion of the trial. No single method is generally accepted as preferred. One used in the past was to exclude subjects from the analyses if they did not complete the study (i.e., *completers' analysis*). This strategy is generally inadvisable because the reason for missing data can be treatment-related and these patients may not adequately represent the study population.

Another common, albeit problematic, strategy is to use the last observation available as the *final *evaluation – usually referred to as last observation carried forward (LOCF). Even though LOCF enables every patient randomized to contribute some observation to the analysis, it can be problematic for the following reasons:

• If the objective of the trial is to detect a treatment effect after a certain duration of treatment (e.g., at 8 weeks), then a comparison that includes only measurements on patients at earlier times or visits is not addressing the original trial objective. The average of patient responses, many of which are at different times or visits, may be uninterpretable.

• LOCF makes an implicit assumption that the patient would sustain the same response seen at an early study visit for the entire duration of the trial. This assumption is untestable and potentially unrealistic.

Some other approaches involve imputation of missing data on a per-patient basis. These strategies try to predict missing outcomes for a patient who has withdrawn from the trial using data from subjects who stayed in the trial and for whom all data have been collected. All of these strategies are imperfect, as they involve strong or weak assumptions about what caused data to be missing, assumptions that usually cannot be verified from the data. If missing data are associated with treatment effect in ways that cannot be predicted from measurements on subjects with complete data, analyses using imputation procedures will be biased. When there are few patients with missing measurements and the frequency of missing data or proportion of patients with missing data is comparable across treatment groups, most approaches will yield similar results. When a higher proportion of patients have missing data, the FDA recommends the use of several different imputation methods (including a worst-case scenario in which missing data are assumed to be unfavorable for those on the investigational treatment and favorable for those in the control group) and an assessment of the consistency of the study results using each method. These analyses will demonstrate the sensitivity of the conclusions to the assumptions made by the different methods.

### 5.5 Interpretation of study results

Because statistical significance can sometimes be achieved for very small changes if a study is large enough, it is tempting to identify an MID as a benchmark for interpreting the clinical importance or relevance of study results. If the MID is truly to be the smallest effect considered meaningful, however, it would be logical to establish the null hypothesis to rule out a difference less than or equal to the MID. This is rarely done, and would have major implications for sample size.

When clinical trials show small mean effect sizes, rather than considering results in terms of an MID, it may be more informative to examine the distribution of responses between treatment groups to more fully characterize the treatment effect and examine the possibility that the mean improvement reflects very different responses in subsets of patients. When only a modest fraction of people respond to a treatment, that fraction may experience meaningful change in the face of a mean effect that is very small. When defining a meaningful change on an individual patient basis (i.e., a responder), that definition is generally larger than the minimum important difference for application to group mean comparisons.

## Glossary

### Claim

A statement of treatment benefit or comparative safety advantage. A claim can appear in any section of a medical product's FDA-approved label or in advertising of prescription drugs.

### Cognitive debriefing

A qualitative research tool used to determine whether concepts and items are understood by patients in the same way that instrument developers intend. Cognitive debriefing interviews involve incorporating follow-up questions in a field test interview to gain a better understanding of how patients interpret questions asked of them.

### Concept

The specific goal of measurement (i.e., the *thing *that is to be measured by a PRO instrument).

### Conceptual framework

The expected relationships of items within a domain and of domains within a PRO concept. The validation process confirms the conceptual framework. When used in a clinical trial, the observed relationships among items and domains will again confirm the conceptual framework.

### Domain

A domain is a discrete concept within a multidomain concept. All the items in a single domain contribute to the measurement of the domain concept.

### Health-related quality of life (HRQL)

A multidomain concept that represents the patient's overall perception of the impact of an illness and its treatment. An HRQL measure captures, at a minimum, physical, psychological (including emotional and cognitive), and social functioning. Claiming a statistical and meaningful improvement in HRQL implies: (1) that the instrument measures all HRQL domains that are important to interpreting change in how the study population feels or functions as a result of treatment; and (2) that improvement was demonstrated in all of the important domains. An HRQL instrument is a particular type of PRO instrument.

### Instrument

A means to capture data (e.g., questionnaire, diary) plus all the information and documentation that supports its use. Generally, that includes clearly defined methods and instructions for administration or responding, a standard format for data collection, and well-documented methods for scoring, analysis, and interpretation of results.

### Item

An individual question, statement, or task that is evaluated by the patient to address a particular concept.

### Minimum important difference (MID)

The amount of difference or change observed in a PRO measure between treatment groups in a clinical trial that will be interpreted as a treatment benefit.

### Patient-reported outcome (PRO)

Any report coming directly from patients (i.e., study subjects) about a health condition and its treatment.

### Quality of life

A general concept that implies an evaluation of the impact of all aspects of life on general well-being. Because this term implies the evaluation of nonhealth-related aspects of life, it is too broad to be considered appropriate for a medical product claim.

### Questionnaire

A set of questions or items shown to a respondent in order to get answers for research purposes.

### Scale

The system of numbers or verbal anchors by which a value or score is derived. Examples include visual analogue scales, Likert scales, and rating scales.

### Score

A number derived from a patient's response to items in a questionnaire. A score is computed based on a prespecified, validated scoring algorithm and is subsequently used in statistical analyses of clinical study results. Scores can be computed for individual items, domains, or concepts, or as a summary of items, domains, or concepts.

### Treatment benefit

An improvement in how a patient survives, feels, or functions as a result of treatment. Measures that do not directly capture the impact of treatment on how a patient survives, feels, or functions are surrogate measures of treatment benefit.

### Validation

The process of assessing a PRO instrument's ability to measure a specific concept or collection of concepts. This ability is described in terms of the instrument's measurement properties that are derived during the validation process. At the conclusion of the process, a set of measurement properties is produced that are specific to the specific population and the specific form and format of the PRO instrument tested. The validation process involves:

• Identifying the concept to be measured

• Assessing the content validity (i.e., being sure the items in the questionnaire cover all important aspects of the concept from the patient perspective)

• Evaluating the proposed scores to be obtained from the instrument

• Defining *a priori *hypotheses of the expected relationships between PRO concepts and other measures

• Testing the hypotheses by reporting the observed correlations among scores

## Availability

For questions regarding this draft document contact Laurie Burke (CDER) 301-796-0700, Toni Stifano (CBER) 301-827-6190, or Sahar Dawisha (CDRH) 301-594-3090.

Additional copies are available from:

Office of Training and Communications, Division of Drug Information, HFD-240, Center for Drug Evaluation and Research, Food and Drug Administration, 5600 Fishers Lane, Rockville, MD 20857, USA

(Tel) 301-827-4573



Office of Communication, Training, and Manufacturers Assistance, HFM-40, Center for Biologics Evaluation and Research, Food and Drug Administration, 1401 Rockville Pike, Rockville, MD 20852-1448

(Tel) 800-835-4709 or 301-827-1800



Office of Communication, Education, and Radiological Programs, Division of Small Manufacturers Assistance, HFZ-220, Center for Devices and Radiological Health, Food and Drug Administration, 1350 Piccard Drive

Rockville, MD 20850-4307, USA

(Tel) Manufacturers Assistance: 800-638-2041 or 301-443-6597

(Tel) International Staff Phone: 301-827-3993

E-mail: dsma@cdrh.fda.gov

Fax: 301-443-8818



## Authors' contributions

This guidance has been prepared by the Office of New Drugs and the Office of Medical Policy in the Center for Drug Evaluation and Research (CDER) in cooperation with the Center for Biologics Evaluation and Research (CBER) and the Center for Devices and Radiological Health (CDRH) at the Food and Drug Administration.

## Appendix

**1**. *Labeling*, as used in this guidance, refers to the medical product description and summary of use, safety, and effectiveness that must be approved by the FDA. See 21 CFR 201.56 and 201.57 for regulations pertaining to prescription drug (including biological drug) labeling. For medical device labeling, see 21 CFR 801. For blood and blood products for transfusion, see 21 CFR 606.122 Instruction Circular.

**2**. For drugs, section 505(d) of the Federal Food, Drug, and Cosmetic Act (the Act) establishes *substantial evidence *as the evidence standard for making conclusions that a drug will have a claimed effect and states that reports of adequate and well-controlled investigations provide the basis for determining whether there is *substantial evidence *to support claims of effectiveness for new drugs. See 21 CFR 314.126 for a description of the characteristics of an adequate and well-controlled investigation. See the guidance for industry *Providing Clinical Evidence of Effectiveness for Human Drug and Biological Products *for considerations concerning the quantity of evidence necessary to meet the *substantial evidence *standard . For medical devices, the Medical Device Amendments of 1976 to the Act established the assurance of safety and effectiveness of medical devices intended for human use. See 21 CFR 860.7 for the evidence used in the determination of safety and effectiveness of a medical device.

**3**. The FDA is specifically asking for comment on the need for, and appropriate standards for, MID definitions applied to PRO instruments used in clinical studies.

**4**. The FDA is specifically asking for comment on the appropriate review standards for the definition of a responder when applied to PRO instruments used in clinical studies to support medical product development.

**5**. For the principal record keeping requirements for clinical investigators and sponsors, see 21 CFR 312.50, 312.58, 312.62, 312.68, 812.140, and 812.145.

**6**. See the draft guidance for industry *Computerized Systems Used in Clinical Trials*. When final, this guidance will supersede the guidance of the same name issued in April 1999 and will represent the FDA's current thinking on this topic. For the most recent version of a guidance, check the CDER guidance Web page at .

**7**. See the guidance for industry *Part 11, Electronic Records; Electronic Signatures – Scope and Application *

**8**. The FDA specifically welcomes comment and additional information that will inform these policies as new electronic PRO technology is developed and used in the medical product development setting.

**9**. See the ICH guidance for industry *E9 Statistical Principles for Clinical Trials *

## Disclaimer

This draft guidance, when finalized, will represent the Food and Drug Administration's (FDA's) current thinking on this topic. It does not create or confer any rights for or on any person and does not operate to bind FDA or the public. You can use an alternative approach if the approach satisfies the requirements of the applicable statutes and regulations. If you want to discuss an alternative approach, contact the FDA staff responsible for implementing this guidance. If you cannot identify the appropriate FDA staff, call the appropriate number listed on the title page of this guidance.

